# Supplementary material for: Interaction patterns and individual dynamics shape the way we move in synchrony
Source: Sci Rep. 2017 Jul 28;7:6846. doi: 10.1038/s41598-017-06559-4 (PMC5533803; doi:10.1038/s41598-017-06559-4)
Supplement: Supplementary file 1 — Supplementary Information [file 41598_2017_6559_MOESM1_ESM.pdf]

Interaction patterns and individual dynamics shape the way we move in synchrony
# - Supplementary Information -

Francesco Alderisio[†], Gianfranco Fiore[†], Robin N. Salesse[‡],
Benoît G. Bardy[‡§] & Mario di Bernardo[†¶] *

[†]Department of Engineering Mathematics, Merchant Venturers Building, University of Bristol, Woodland Road, Clifton, Bristol BS8 1UB, United Kingdom (f.alderisio@bristol.ac.uk, gianfranco.fiore@bristol.ac.uk, m.dibernardo@bristol.ac.uk)

[‡]EuroMov, Montpellier University, 700 Avenue du Pic Saint-Loup, 34090 Montpellier, France (benoit.bardy@umontpellier.fr, salesse.robin@gmail.com)

[§]Institut Universitaire de France, 1 rue Descartes, 75231 Paris Cedex 05, France

[¶]Department of Electrical Engineering and Information Technology, University of Naples Federico II, Via Claudio 21, 80125 Naples, Italy (mario.dibernardo@unina.it)

1 Experimental protocol

Participants were asked to sit in a circle on plastic chairs (Supplementary Fig. 1a) and move their preferred hand as smoothly as possible back and forth (that is, away from and back to their torso) on a plane parallel to the floor, along a direction required to be straight.

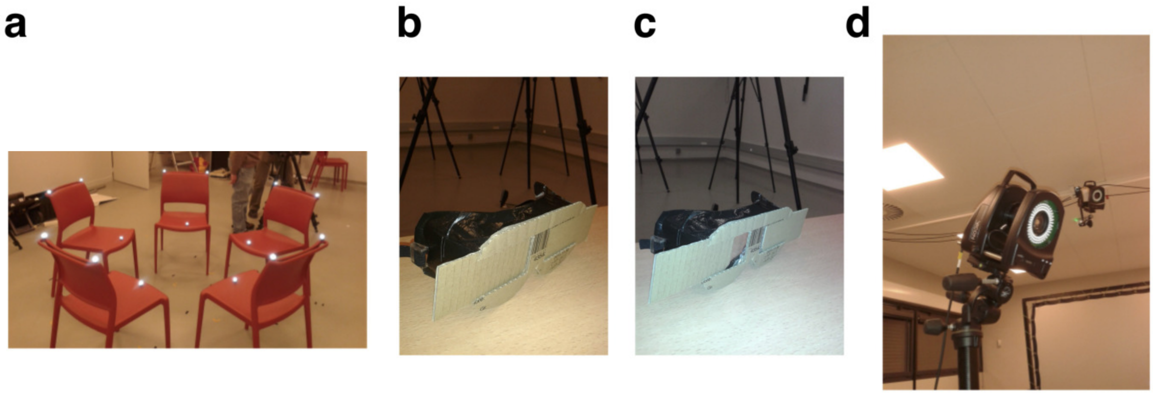

Supplementary Figure 1: **Experimental platform used for the experiments.** (**a**) Plastic chairs with position markers. (**b**) Shut goggles: players are deprived of their own sight. (**c**) Open goggles: by appropriately sliding the mobile cardboard on the fixed one, players can modify their own field of view. (**d**) Eight cameras are employed to track the position of the hand of each player.

Different interaction patterns (also referred to in the text as interaction structure or topology) were implemented by asking each of the players to take into consideration the motion of only a designated subset of the other players. In order to physically implement these different interconnections, the field of view (FOV) of some players was reduced by means of *ad hoc* goggles. In particular, black duct tape was wrapped around such goggles in order to mask the peripheral FOV of the players on both sides. In addition, two cardboards, one of which was mobile, were appropriately glued on the goggles so as to restrict the FOV angle of each player by adjusting the position of the sliding cardboard (Supplementary Figs 1b and 1c). In such a way it was possible to implement different visual pairings among the players.

- **Complete graph** (Figs 2a,e in the main text): participants sat in a circle facing each other without wearing the goggles. They were asked to keep their gaze focused on the middle of the circle in order to see the movements of all the others.
- **Ring graph** (Figs 2b,f in the main text): participants sat in a circle facing each other while wearing the goggles. Each player was asked to see the hand motion of only two others, called *partners*. The goggles allowed participants to focus their gaze on the motion of their only two designated *partners*.
- **Path graph** (Figs 2c,f in the main text): similar to the Ring graph configuration, but two participants, defined as *external*, were asked to see the hand motion of only one *partner* (not the same). This was realised by removing the visual coupling between any pair of participants in the Ring graph configuration.
- **Star graph** (Figs 2d,g in the main text): all participants but one sat side by side facing the remaining participant while wearing the goggles. The former, defined as *peripheral* players, were asked to focus their gaze on the motion of the latter, defined as *central* player, who in turn was asked to see the hand motion of all the others.

2 Data analysis

In order to detect and analyse the three-dimensional position of the participants' hands, eight infrared cameras (Nexus MX13 Vicon System ©) were located around the experimental room (Supplementary Fig. 1d). Despite each of them achieving full frame synchronisation up to $350Hz$, data was recorded with a sampling frequency of $100Hz$, with an estimated error of $0.01mm$ for each coordinate.

In order for the cameras to detect the position of each player's hand, circular markers were attached on top of their index fingers; such positions were provided as triplets of (x, y, z) coordinates in a Cartesian frame of reference (Supplementary Fig. 2, black axes). In rare occasions (0.54% of the total number of data points for Group 1, never for Group 2) it was necessary to deprive these trajectories of possible undesired spikes caused by the cameras not being able to appropriately detect the position of the markers for the whole duration of the trial. As for Group 1, spikes were found in:

- 1 trajectory of Player 2 in the Ring graph topology;
- 2 trajectories of Player 2 in the Path graph topology;
- 4 trajectories of Player 2 and 8 trajectories of Player 7 in the Star graph topology.

After removing possible spikes, classical interpolation techniques were used to fill the gap previously occupied by the spikes themselves. Besides, since the players' positions were provided as triplets of (x, y, z) coordinates but essentially the motion of each player could be described as a one-dimensional movement, it was necessary to perform *principal component analysis* (PCA) on the collected trajectories to find such direction, which turns out to correspond to the x_{PCA} axis (Supplementary Fig. 2, in red).

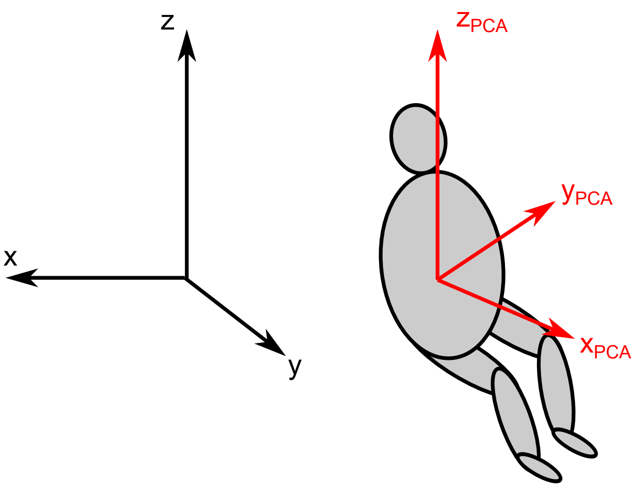

Supplementary Figure 2: Cartesian frame of reference (x, y, z) and principal components $(x_{PCA}, y_{PCA}, z_{PCA})$. The axes x and y lie on a plane which is parallel to the ground, while the z axis is orthogonal to it. The axes x_{PCA}, y_{PCA} and z_{PCA} individuate the principal components: x_{PCA} is the direction where most of the movement takes place.

PCA was applied to the collected players' trajectories defined by x and y, since the hand motion took place mostly in the (x, y) plane so that the z-coordinate could be neglected, obtaining the principal components x_{PCA} and y_{PCA}. Since the motion along the component y_{PCA} turns out to be negligible compared to that along x_{PCA}, it is possible to further assume that the motion of each player is one-dimensional (Supplementary Fig. 3). For this reason, after removing possible spikes, all the data collected in the experiments underwent PCA and then only the first principal component, namely x_{PCA}, was considered for further analysis.

3 Parameterisation and initialisation of the mathematical model

Given the oscillatory nature of the task participants were required to perform, we used a network of heterogeneous nonlinearly coupled Kuramoto oscillators as mathematical model to capture the experimental observations

$$\dot{\theta}_k = \omega_k + \frac{c}{N} \sum_{h=1}^{N} a_{kh} \sin\left(\theta_h - \theta_k\right), \qquad k = 1, 2, \ldots, N \tag{1}$$

The values of the players' natural oscillation frequencies ω_k were estimated by considering the M eyes-closed trials ($M = 16$ for Group 1, and $M = 10$ for Group 2). Specifically, we evaluated the

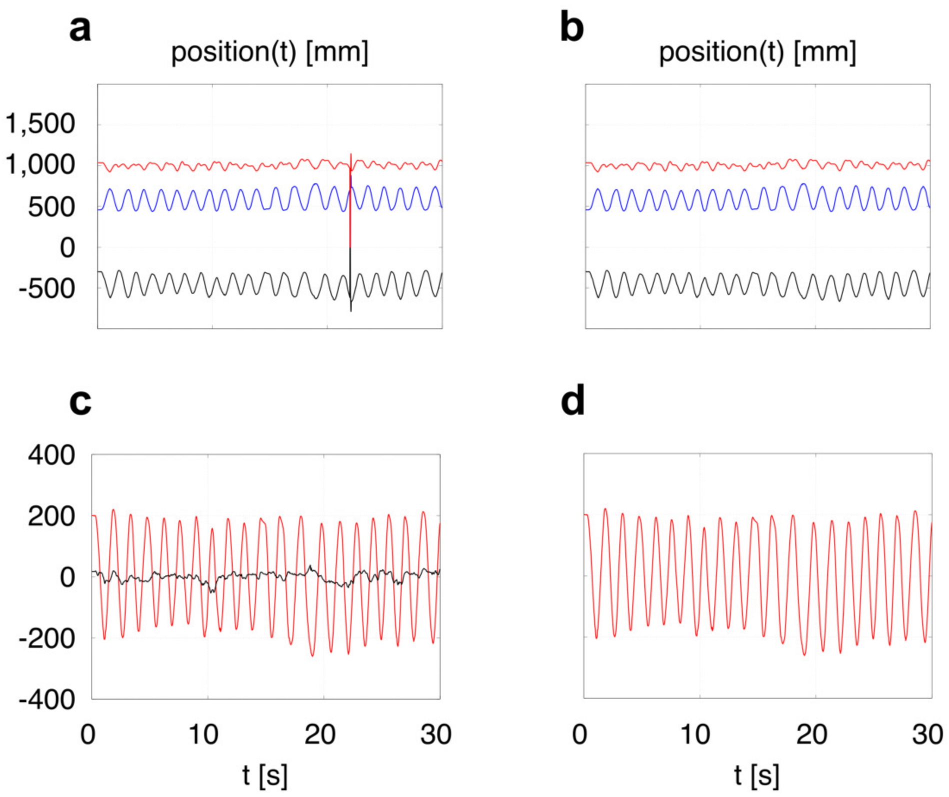

Supplementary Figure 3: Pre-analysis for the hand motion of a given participant. (**a**) Original trajectories defined by (x, y, z), respectively represented in blue, black and red, after recording the hand motion of each participant. (**b**) Trajectories (x, y, z), respectively represented in blue, black and red, after removing the spikes. (**c**) Trajectories (x_{PCA}, y_{PCA}), respectively represented in red and black, after PCA analysis applied onto x and y. (**d**) One-dimensional trajectory, defined by x_{PCA} (in red), used for further analysis.

fundamental harmonic of each player's motion from the Fourier transform of their position trajectory, thus obtaining M values for each participant. For our simulations, we assumed that each oscillation frequency ω_k of Supplementary equation (1) was a time-varying quantity, randomly extracted from a Gaussian distribution whose mean $\mu(\omega_k)$ and standard deviation $\sigma(\omega_k)$ are evaluated from the M aforementioned values collected for each human participant (see Supplementary Fig. 4 and Supplementary Tables 1 and 2 for more details). Indeed, the Kolmogorov-Smirnov test decision for the null hypothesis that the experimental data comes from a normal distribution was performed, and such test always failed to reject the null hypothesis at the 5% significance level.

Supplementary Table 1: Mean value and standard deviation, over the total number of eyes-closed trials, of the players' natural oscillation frequencies – Group 1.

**Player**	$\mu(\omega_k)$	$\sigma(\omega_k)$
1	4.2568	0.3941
2	4.3143	0.3492
3	4.6691	0.3999
4	4.2951	0.3543
5	4.3623	0.3406
6	2.9433	0.6609
7	4.2184	0.3314

The mean value of the frequencies is indicated with $\mu(\omega_k)$, while their standard deviation is indicated with $\sigma(\omega_k)$, $\forall k \in [1, N]$.

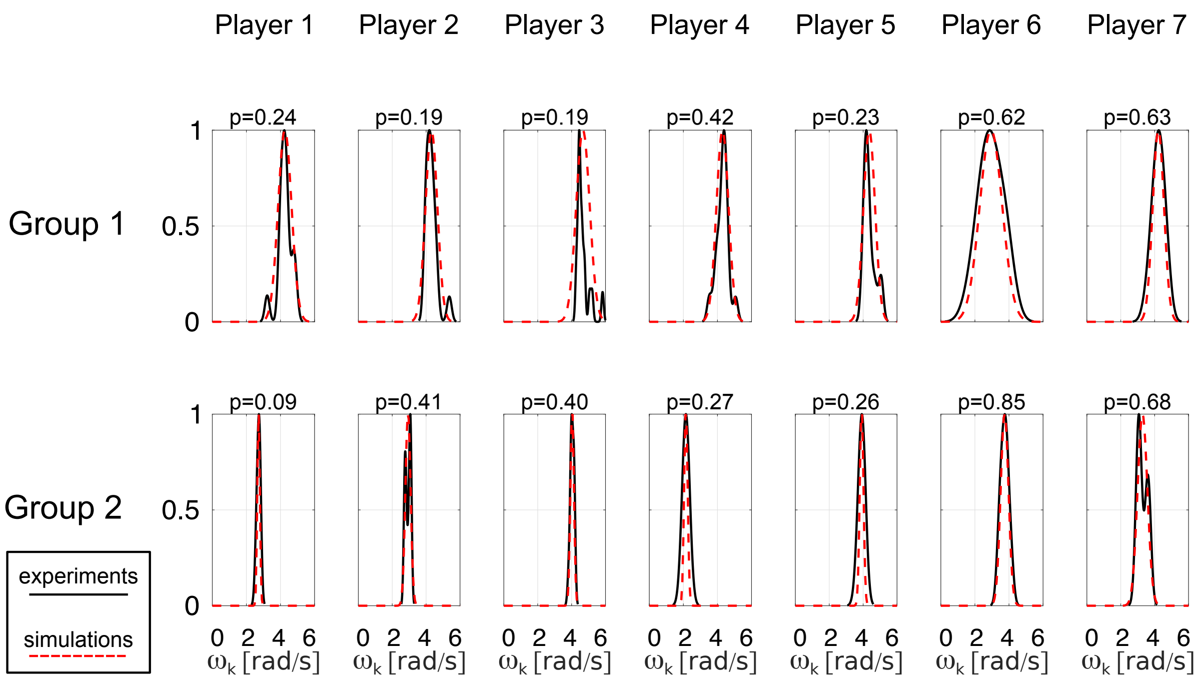

Supplementary Figure 4: Probability distribution function of natural oscillation frequencies ω_k. The probability distribution functions evaluated from the M values of ω_k obtained experimentally in the eyes-closed trials are represented as black solid lines, whereas the fitted normal distributions used in the numerical simulations are represented as red dashed lines. The null hypothesis that the experimental data comes from a normal distribution was tested. Such hypothesis could never be rejected, as specified by a p-value always greater than 5%. The top row refers to players of Group 1, while the bottom row to those of Group 2, whereas each column refers to a different player in the group.

Supplementary Table 2: Mean value and standard deviation, over the total number of eyes-closed trials, of the players' natural oscillation frequencies – Group 2.

**Player**	$\mu(\omega_k)$	$\sigma(\omega_k)$
1	2.7151	0.0741
2	2.9299	0.1525
3	4.0344	0.1035
4	2.1476	0.1023
5	3.9117	0.1085
6	3.7429	0.2309
7	3.2827	0.2911

The mean value of the frequencies is indicated with $\mu(\omega_k)$, while their standard deviation is indicated with $\sigma(\omega_k)$, $\forall k \in [1, N]$.

Furthermore, as confirmation of the fact that players in both groups exhibited time-varying natural oscillation frequencies ω_k, we also computed the Hilbert transform of each position trajectory $x_k(t)$ collected in the M eyes-closed trials, then we evaluated its first time derivative, and finally observed that such derivative is a time-varying signal (Supplementary Figs 5 and 6). Note that the Hilbert and the Fourier transform methods lead to consistent results for the mean values and the standard deviations of the M average values of ω_k over time, respectively for each kth player.

By defining $\tilde{\omega} := [\mu(\omega_1)\ \ \mu(\omega_2)\ \ \ldots\ \mu(\omega_7)]^T \in \mathbb{R}^7$, it is possible to obtain the coefficient of variation:

$$c_v := \frac{\sigma(\tilde{\omega})}{\mu(\tilde{\omega})} \tag{2}$$

which is equal to $c_{v_1} \simeq 0.13$ for Group 1 and $c_{v_2} \simeq 0.21$ for Group 2. Such coefficient of variations

quantify the overall dispersions of the natural oscillation frequencies of the players, respectively for the two groups. Analogously, it is possible to define the individual coefficient of variation:

$$c_v(\omega_k) := \frac{\sigma(\omega_k)}{\mu(\omega_k)} \tag{3}$$

as a measure of the individual variability of the natural oscillation frequency of each kth player.

As for the coupling strength c, we found that setting the same constant value for all the topologies under investigation captures well the experimental observations (see Supplementary Section 4 below). As for the initial values of the phases, since before starting any trial all the human players were asked to completely extend their arm so that the first movement would be pulling their arm back towards their torso from the same initial conditions, we set $\theta_k(0) = \frac{\pi}{2}$ for all the nodes, trials and topologies.

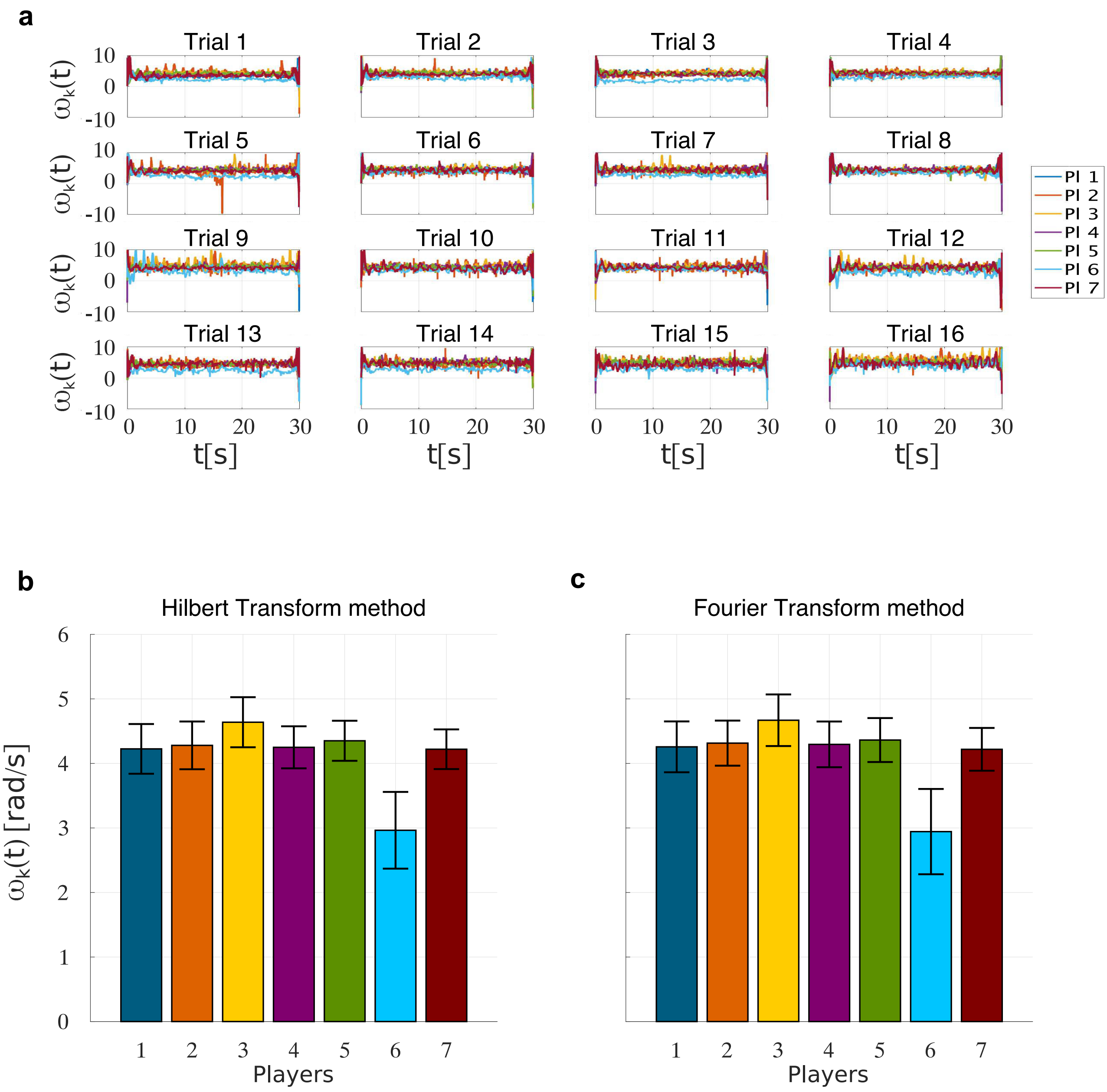

Supplementary Figure 5: **Natural oscillation frequencies** ω_k **– Group 1.** For all the $M = 16$ eyes-closed trials, the angular velocity $\omega_k(t)$ of each kth player, estimated through the Hilbert transform method, is a time-varying signal (**a**). Mean values (colour-coded bars) and standard deviations (black vertical bars) of the M average values of ω_k over time are represented for the Hilbert transform method (**b**) and the Fourier transform method (**c**), respectively. Different colours refer to different players.

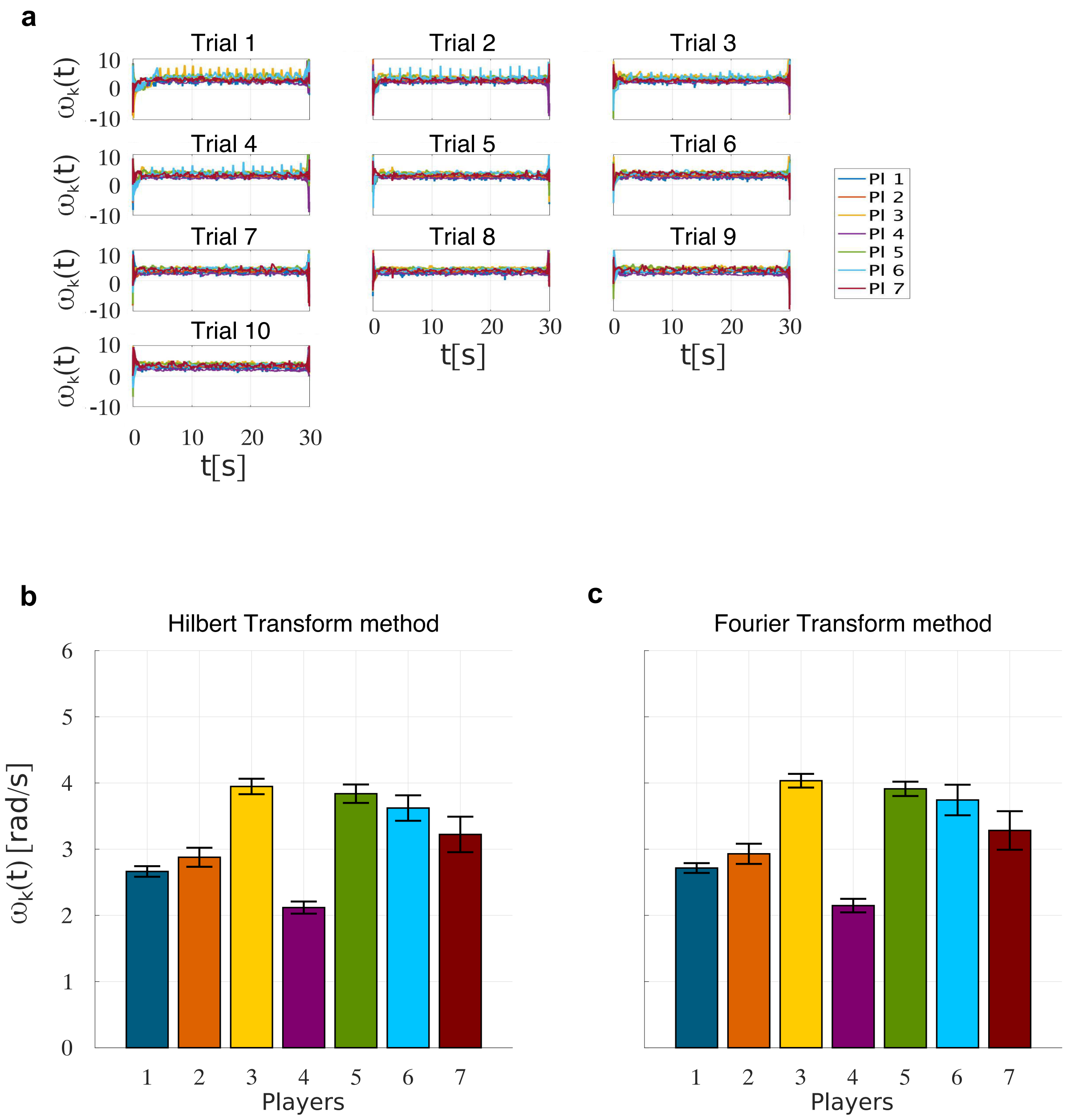

Supplementary Figure 6: **Natural oscillation frequencies** ω_k − **Group 2.** For all the $M = 10$ eyes-closed trials, the angular velocity $\omega_k(t)$ of each kth player, estimated through the Hilbert transform method, is a time-varying signal (**a**). Mean values (colour-coded bars) and standard deviations (black vertical bars) of the M average values of ω_k over time are represented for the Hilbert transform method (**b**) and the Fourier transform method (**c**), respectively. Different colours refer to different players.

4 Group synchronisation indices

For each of the two groups we show the group synchronisation indices obtained experimentally and numerically by simulating the model proposed in Supplementary equation (1), with two different values of coupling strength c set as described in *Methods* of the main text (Supplementary Table 3 for Group 1, and Supplementary Table 4 for Group 2).

Supplementary Table 3: **Mean value** $\mu(\rho_g)$ **and standard deviation** $\sigma(\rho_g)$ **over time of the *group synchronisation index*, averaged over the total number of eyes-open trials – Group 1,** $c_{v_1} = 13\%$**.**

**Topology**	**Experiments**	**Simulations,** $c = 1.25$	**Simulations,** $c = 4.40$
Complete graph	0.9556 ± 0.0414	0.9462 ± 0.0772	0.9999 ± 0.0003
Ring graph	0.7952 ± 0.1532	0.8193 ± 0.1048	0.9575 ± 0.0740
Path graph	0.8661 ± 0.1173	0.7446 ± 0.1309	0.8302 ± 0.1630
Star graph	0.9285 ± 0.0753	0.8730 ± 0.0993	0.8255 ± 0.1663

This table shows $\mu(\rho_g) \pm \sigma(\rho_g)$ for both experimental and simulation results.

Supplementary Table 4: **Mean value** $\mu(\rho_g)$ **and standard deviation** $\sigma(\rho_g)$ **over time of the *group synchronisation index*, averaged over the total number of eyes-open trials – Group 2,** $c_{v_2} = 21\%$**.**

**Topology**	**Experiments**	**Simulations,** $c = 4.40$	**Simulations,** $c = 1.25$
Complete graph	0.9559 ± 0.0508	0.9999 ± 0.0005	0.9339 ± 0.0862
Ring graph	0.8358 ± 0.1130	0.8633 ± 0.1460	0.4799 ± 0.2155
Path graph	0.7534 ± 0.1766	0.7265 ± 0.2293	0.4756 ± 0.2061
Star graph	0.9759 ± 0.0274	0.8624 ± 0.1158	0.5450 ± 0.1749

This table shows $\mu(\rho_g) \pm \sigma(\rho_g)$ for both experimental and simulation results.

5 Dyadic synchronisation indices

For both Group 1 and Group 2, we show mean values and standard deviations of $\rho_{d_{h,k}}$ over the 10 eyes-open trials of each topology. In particular, if we denote with $\rho^{(l)}_{d_{h,k}}$ the value of the *dyadic synchronisation index* $\rho_{d_{h,k}}$ in the l-th trial of a certain topology, the mean value over the total number of trials is given by

$$\rho_{\mu,hk} = \frac{1}{10}\sum_{l=1}^{10} \rho^{(l)}_{d_{h,k}} \tag{4}$$

Similarly, the standard deviation is given by

$$\rho_{\sigma,hk} = \sqrt{\frac{1}{10}\sum_{l=1}^{10} \left(\rho^{(l)}_{d_{h,k}} - \rho_{\mu,hk}\right)^2} \tag{5}$$

For both Group 1 and Group 2, mean values and standard deviations of the *dyadic synchronisation index* are shown for all the pairs in the four implemented topologies (Supplementary Fig. 7). For the sake of clarity, all values of $\rho_{\mu,hk}$ and $\rho_{\sigma,hk}$ are expressed as percentiles (multiplied by 100). In most cases (99% for Group 1 and 94% for Group 2) the highest mean values of the dyadic synchronisation indices are observed for the visually connected dyads (represented in bold in Supplementary Fig. 7), meaning that players managed to maximise synchronisation with those they were visually coupled with.

In particular:

- Complete graph: all means $\rho_{\mu,hk}$ are higher than 0.82 for Group 1 and 0.86 for Group 2.

- Ring graph: for each player of Group 1 the highest values of $\rho_{\mu,hk}$ are obtained with respect to the two agents that player was asked to be topologically connected with, whereas for each player of Group 2 at least either of the two values of $\rho_{\mu,hk}$ related to her/his *partners* turns out to be the highest, and that related to the other *partner* is either the second highest (nodes 2, 4 and 7), the third highest (nodes 1, 3 and 6) or the fourth highest (node 5).

- Path graph: remarks analogous to those of the Ring graph configuration can be made. The only exception is node 4 for Group 1, where $\rho_{\mu,43} < \rho_{\mu,46}$ (however the two values are still close to each other, as $\rho_{\mu,43} = \rho_{\mu,34} = 0.82$ and $\rho_{\mu,46} = \rho_{\mu,64} = 0.84$), and node 3 for Group 2, where $\rho_{\mu,34} = \rho_{\mu,43} = 0.78$ is lower than $\rho_{\mu,31} = \rho_{\mu,13} = 0.89$. It is also worth pointing out how, for both groups, consistently with the implemented network of interactions, the mean values $\rho_{\mu,17} = \rho_{\mu,71}$ are lower than those corresponding to the Ring graph configuration, which is a consequence of removing the visual coupling between the *external* agents 1 and 7.

- Star graph: for each *peripheral* player of both the groups, the highest values of $\rho_{\mu,hk}$ are obtained with respect to Player 3 (*central* player).

As for the standard deviations $\rho_{\sigma,hk}$, in most cases (86% for Group 1 and 89% for Group 2) the lowest values are observed for the topologically connected dyads (represented in bold in Supplementary Fig. 7), which confirms the robustness of the interactions between visually coupled pairs.

Topology	Group 1	Group 2

Complete graph

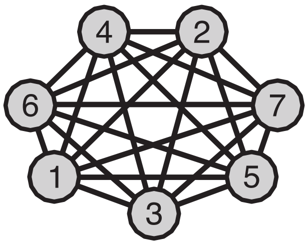

Group 1

	1	2	3	4	5	6	7
1	-	**91_{5}**	**94_{4}**	**94_{4}**	**90_{7}**	**86_{13}**	**93_{4}**
2	**91_{5}**	-	**91_{6}**	**91_{7}**	**87_{8}**	**82_{12}**	**91_{3}**
3	**94_{4}**	**91_{6}**	-	**95_{2}**	**94_{2}**	**85_{13}**	**94_{3}**
4	**94_{4}**	**91_{7}**	**95_{2}**	-	**94_{3}**	**88_{9}**	**94_{2}**
5	**90_{7}**	**87_{8}**	**94_{2}**	**94_{3}**	-	**84_{12}**	**91_{4}**
6	**86_{13}**	**82_{12}**	**85_{13}**	**88_{9}**	**84_{12}**	-	**85_{10}**
7	**93_{4}**	**91_{3}**	**94_{3}**	**94_{2}**	**91_{4}**	**85_{10}**	-

Group 2

	1	2	3	4	5	6	7
1	-	**89_{10}**	**92_{11}**	**94_{3}**	**92_{11}**	**92_{11}**	**91_{9}**
2	**89_{10}**	-	**92_{5}**	**86_{14}**	**90_{6}**	**89_{14}**	**92_{4}**
3	**92_{11}**	**92_{5}**	-	**92_{11}**	**93_{4}**	**91_{13}**	**92_{6}**
4	**94_{3}**	**86_{14}**	**92_{11}**	-	**91_{10}**	**92_{11}**	**88_{12}**
5	**92_{11}**	**90_{6}**	**93_{4}**	**91_{10}**	-	**92_{13}**	**92_{5}**
6	**92_{11}**	**89_{14}**	**91_{13}**	**92_{11}**	**92_{13}**	-	**89_{15}**
7	**91_{9}**	**92_{4}**	**92_{6}**	**88_{12}**	**92_{5}**	**89_{15}**	-

Ring graph

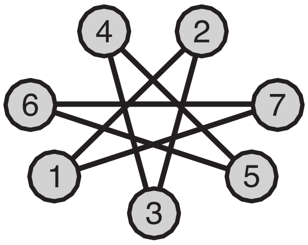

Group 1

	1	2	3	4	5	6	7
1	-	**74_{28}**	59_{31}	59_{27}	47_{26}	65_{28}	**75_{24}**
2	**74_{28}**	-	**72_{28}**	64_{29}	51_{28}	51_{29}	56_{26}
3	59_{31}	**72_{28}**	-	**84_{18}**	67_{27}	60_{35}	59_{29}
4	59_{27}	64_{29}	**84_{18}**	-	**83_{20}**	69_{29}	65_{23}
5	47_{26}	51_{28}	67_{27}	**83_{20}**	-	**78_{25}**	63_{23}
6	65_{28}	51_{29}	60_{35}	69_{29}	**78_{25}**	-	**81_{21}**
7	**75_{24}**	56_{26}	59_{29}	65_{23}	63_{23}	**81_{21}**	-

Group 2

	1	2	3	4	5	6	7
1	-	**77_{23}**	63_{28}	61_{29}	65_{29}	78_{16}	**86_{12}**
2	**77_{23}**	-	**79_{26}**	75_{27}	73_{28}	68_{24}	73_{20}
3	63_{28}	**79_{26}**	-	**91_{14}**	81_{21}	61_{27}	64_{22}
4	61_{29}	75_{27}	**91_{14}**	-	**83_{20}**	60_{29}	61_{27}
5	65_{29}	73_{28}	81_{21}	**83_{20}**	-	**69_{31}**	68_{26}
6	78_{16}	68_{24}	61_{27}	60_{29}	**69_{31}**	-	**91_{4}**
7	**86_{12}**	73_{20}	64_{22}	61_{27}	68_{26}	**91_{4}**	-

Path graph

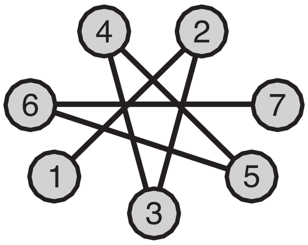

Group 1

	1	2	3	4	5	6	7
1	-	**93_{3}**	82_{7}	69_{17}	60_{27}	57_{28}	55_{28}
2	**93_{3}**	-	**89_{5}**	75_{16}	65_{24}	62_{26}	60_{27}
3	82_{7}	**89_{5}**	-	**82_{15}**	73_{20}	68_{22}	64_{25}
4	69_{17}	75_{16}	**82_{15}**	-	**89_{12}**	84_{13}	79_{16}
5	60_{27}	65_{24}	73_{20}	**89_{12}**	-	**92_{3}**	86_{8}
6	57_{28}	62_{26}	68_{22}	84_{13}	**92_{3}**	-	**94_{3}**
7	55_{28}	60_{27}	64_{25}	79_{16}	86_{8}	**94_{3}**	-

Group 2

	1	2	3	4	5	6	7
1	-	**96_{3}**	89_{6}	73_{22}	56_{30}	41_{19}	36_{17}
2	**96_{3}**	-	**93_{5}**	73_{26}	58_{32}	43_{20}	37_{18}
3	89_{6}	**93_{5}**	-	**78_{25}**	61_{31}	44_{21}	38_{19}
4	73_{22}	73_{26}	**78_{25}**	-	**74_{21}**	49_{19}	41_{18}
5	56_{30}	58_{32}	61_{31}	**74_{21}**	-	**66_{14}**	59_{14}
6	41_{19}	43_{20}	44_{21}	49_{19}	**66_{14}**	-	**88_{8}**
7	36_{17}	37_{18}	38_{19}	41_{18}	59_{14}	**88_{8}**	-

Star graph

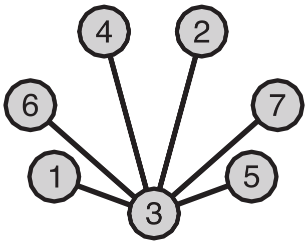

Group 1

	1	2	3	4	5	6	7
1	-	91_{5}	**93_{4}**	92_{5}	91_{3}	86_{13}	72_{25}
2	91_{5}	-	**92_{2}**	91_{2}	89_{3}	82_{16}	73_{24}
3	**93_{4}**	**92_{2}**	-	**96_{1}**	**95_{2}**	**87_{16}**	**76_{24}**
4	92_{5}	91_{2}	**96_{1}**	-	94_{2}	83_{16}	74_{23}
5	91_{3}	89_{3}	**95_{2}**	94_{2}	-	84_{15}	73_{25}
6	86_{13}	82_{16}	**87_{16}**	83_{16}	84_{15}	-	69_{24}
7	72_{25}	73_{24}	**76_{24}**	74_{23}	73_{25}	69_{24}	-

Group 2

	1	2	3	4	5	6	7
1	-	94_{4}	**97_{1}**	96_{1}	97_{1}	94_{2}	94_{2}
2	94_{4}	-	**95_{4}**	94_{5}	94_{5}	90_{6}	92_{5}
3	**97_{1}**	**95_{4}**	-	**97_{1}**	**98_{1}**	**94_{2}**	**95_{2}**
4	96_{1}	94_{5}	**97_{1}**	-	97_{1}	93_{4}	94_{2}
5	97_{1}	94_{5}	**98_{1}**	97_{1}	-	94_{2}	94_{2}
6	94_{2}	90_{6}	**94_{2}**	93_{4}	94_{2}	-	91_{3}
7	94_{2}	92_{5}	**95_{2}**	94_{2}	94_{2}	91_{3}	-

Supplementary Figure 7: Mean values and standard deviations, over the total number of eyes-open trials, of the *dyadic synchronisation index* obtained experimentally. Each row corresponds to one of the four implemented interaction patterns: topology representation (left column), indices for Group 1 (central column) and Group 2 (right column). Mean values and standard deviations (as subscripts) of $\rho_{d_{h,k}}$ are represented as percentiles for all the pairs of each topology and for both the groups, with bold values referring to pairs who were visually coupled in the experiments (i.e., there exists a link between the two agents in the respective topology representation).

6 ANOVA tables

Supplementary Table 5: 2(Group) X 4(Topology) Mixed ANOVA – individual synchronisation indices ρ_k in the experiments

**Independent variables**	**Degrees of freedom**	F**-value**	p**-value**	η^2
Group	$(1, 12)$	0.053	0.821	0.004
Topology	$(1.648, 19.779)$	29.447	$\simeq 0$	0.710
Group * Topology	$(1.648, 19.779)$	3.908	0.044	0.246

Supplementary Table 6: Post-hoc pairwise comparisons – individual synchronisation indices ρ_k in the experiments – Group 1

**Topologies**	Complete graph	Ring graph	Path graph	Star graph
Complete graph	–	$\simeq 0$	0.146	0.929
Ring graph	$\simeq 0$	–	0.852	$\simeq 0$
Path graph	0.146	0.852	–	0.564
Star graph	0.929	$\simeq 0$	0.564	–

Supplementary Table 7: Post-hoc pairwise comparisons – individual synchronisation indices ρ_k in the experiments – Group 2

**Topologies**	Complete graph	Ring graph	Path graph	Star graph
Complete graph	–	$\simeq 0$	0.002	1
Ring graph	$\simeq 0$	–	0.792	$\simeq 0$
Path graph	0.002	0.792	–	0.001
Star graph	1	$\simeq 0$	0.001	–

Supplementary Table 8: 2(Group) X 4(Topology) Mixed ANOVA – individual synchronisation indices ρ_k in the numerical simulations

**Independent variables**	**Degrees of freedom**	F**-value**	p**-value**	η^2
Group	$(1, 12)$	0.031	0.862	0.003
Topology	$(3, 36)$	5.946	$\simeq 0$	0.331
Group * Topology	$(3, 36)$	0.163	0.920	0.013

Supplementary Table 9: Post-hoc pairwise comparisons – individual synchronisation indices ρ_k in the numerical simulations

**Topology (A)**	**Topology (B)**	**Mean Difference (A-B)**	**p-value**
Complete graph	Ring graph	0.149	0.010
	Path graph	0.254	0.017
	Star graph	0.112	0.370
Ring graph	Complete graph	-0.149	0.010
	Path graph	0.104	0.795
	Star graph	-0.038	1
Path graph	Complete graph	-0.254	0.017
	Ring graph	-0.104	0.795
	Star graph	-0.142	0.706
Star graph	Complete graph	-0.112	0.370
	Ring graph	0.038	1
	Path graph	0.142	0.706

Supplementary Table 10: 2(Data origin) X 4(Topology) Mixed ANOVA – individual synchronisation indices ρ_k in experiments and numerical simulations – Group 1

**Independent variables**	**Degrees of freedom**	**F-value**	**p-value**	η^2
Data origin	$(1, 12)$	0.206	0.658	0.017
Topology	$(1.523, 18.272)$	5.419	0.020	0.311
Data origin * Topology	$(1.523, 18.272)$	0.893	0.400	0.069

Supplementary Table 11: Main effects of Topology on individual synchronisation indices ρ_k in experiments and numerical simulations – Group 1

**Topologies**	Complete graph	Ring graph	Path graph	Star graph
Complete graph	–	0.001	0.174	1.000
Ring graph	0.001	–	1.000	0.003
Path graph	0.174	1.000	–	0.636
Star graph	1.000	0.003	0.636	–

Supplementary Table 12: 2(Data origin) X 4(Topology) Mixed ANOVA – individual synchronisation indices ρ_k in experiments and numerical simulations – Group 2

**Independent variables**	**Degrees of freedom**	**F-value**	**p-value**	η^2
Data origin	$(1, 12)$	0.619	0.447	0.049
Topology	$(1.875, 22.504)$	12.406	$\simeq 0$	0.508
Data origin * Topology	$(1.875, 22.504)$	1.606	0.223	0.118

Supplementary Table 13: Main effects of Topology on individual synchronisation indices ρ_k in experiments and numerical simulations – Group 2

**Topologies**	Complete graph	Ring graph	Path graph	Star graph
Complete graph	–	$\simeq 0$	$\simeq 0$	0.926
Ring graph	$\simeq 0$	–	0.390	0.352
Path graph	$\simeq 0$	0.390	–	0.069
Star graph	0.926	0.352	0.069	–

7 Group synchronisation trend over time

Supplementary Fig. 8 shows the trend over time of the group synchronisation index $\rho_g(t)$ observed in the experiments, as well as in the numerical simulations, for all the eyes-open trials and topologies of Group 1, whereas Supplementary Fig. 9 shows that of Group 2.

It is possible to appreciate that the proposed mathematical model succeeds in replicating the feature observed experimentally that there is no clear shift between transient (low time-varying) and steady state (high constant) values for the group synchronisation index $\rho_g(t)$, and that more noticeable oscillations are observed in the Ring and Path graphs. Furthermore, note how both in the experiments and in the simulations:

- for Group 1 (Supplementary Fig. 8), $\rho_g(t)$ never achieves a constant value at steady state but exhibits persistent oscillations (less noticeable in the Complete graph);
- for Group 2 (Supplementary Fig. 9), $\rho_g(t)$ exhibits higher oscillations in Ring and Path graphs, whereas lower peaks are observed in the Star graph and almost constant steady state values in the Complete graph.

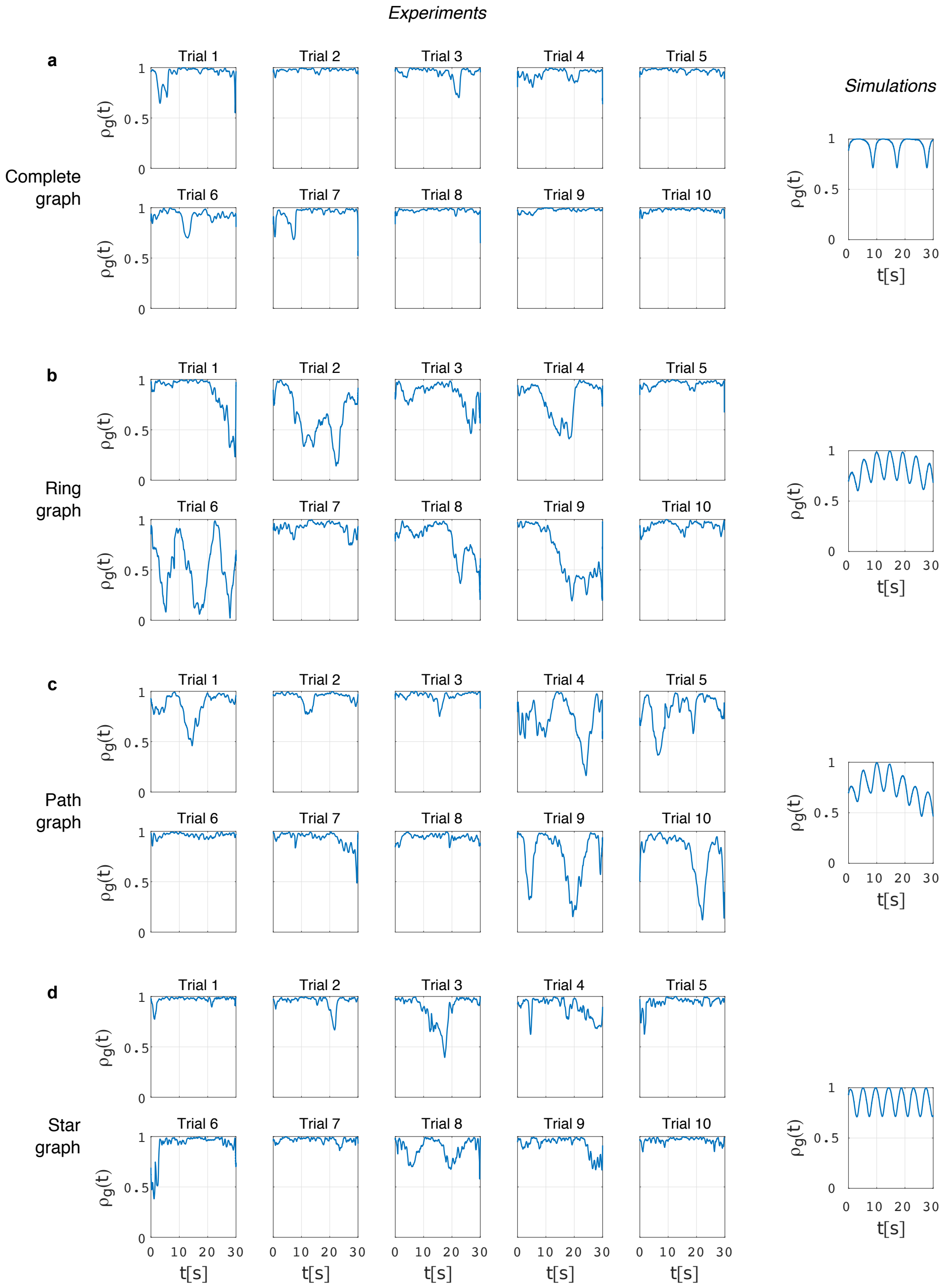

Supplementary Figure 8: **Trend over time of group synchronisation index $\rho_g(t)$ for each trial and topology, both for experiments and numerical simulations – Group 1.** (**a**) Complete graph, (**b**) Ring graph, (**c**) Path graph, (**d**) Start Graph. For each topology, the ten panels on the left show the trend of $\rho_g(t)$ observed experimentally in the ten eyes-open trials, respectively, whereas the panel on the right shows a typical trend of $\rho_g(t)$ obtained numerically for that topology.

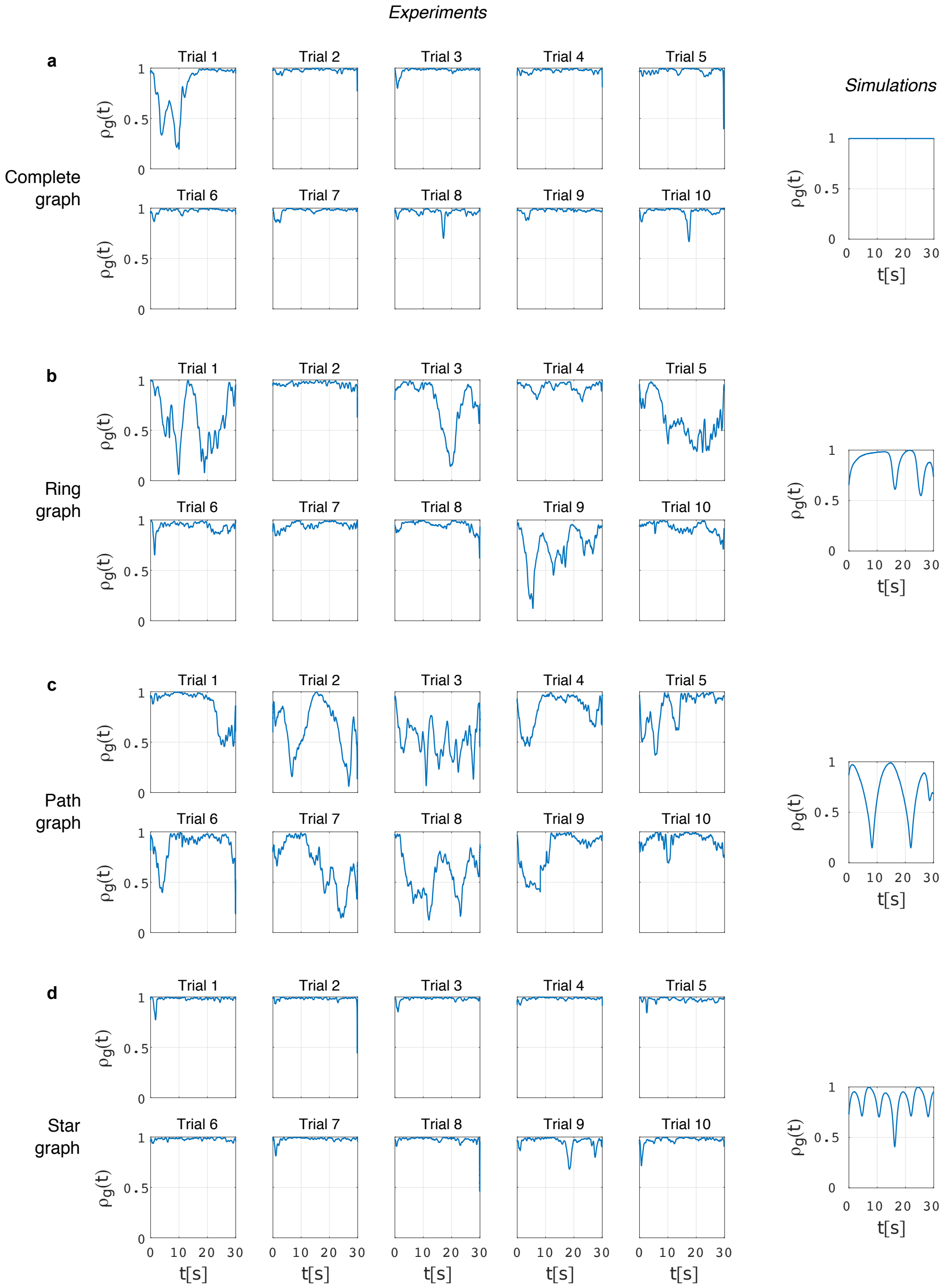

Supplementary Figure 9: Trend over time of group synchronisation index $\rho_g(t)$ for each trial and topology, both for experiments and numerical simulations – Group 2. (**a**) Complete graph, (**b**) Ring graph, (**c**) Path graph, (**d**) Start Graph. For each topology, the ten panels on the left show the trend of $\rho_g(t)$ observed experimentally in the ten eyes-open trials, respectively, whereas the panel on the right shows a typical trend of $\rho_g(t)$ obtained numerically for that topology.

8 Additional model predictions

In order to address the issues raised in *Remark 1* of the main text, in this section we show further results obtained numerically by simulating a network of heterogeneous Kuramoto oscillators. Specifically, the proposed model predicts that:

1. unlike the intra-individual variability of oscillation frequencies $\sigma(\omega_k)$, the overall dispersion c_v has a significant effect on the coordination levels of the group members;
2. the location of the link getting removed from a Ring graph to form a Path graph does not have a significant effect on the coordination levels of its members;
3. different selections of the central node in a Star graph do not have a significant effect on the coordination levels of its members.

8.1 Effects of overall dispersion and intra-individual variability of the natural oscillation frequencies

Firstly, we considered four different groups of $N = 7$ heterogeneous Kuramoto oscillators. In order to isolate the effects of the overall dispersion c_v, the individual standard deviations of the natural oscillation frequencies were not varied across nodes and groups ($\sigma(\omega_k) = 0.25\ \forall k \in [1, N]$), and all the agents in each of the four groups were connected over a Complete graph topology (so that every node is connected to all the others, and no effects of particular topological structures or symmetries are expected). Therefore, the groups differed only for the value of their overall dispersion, respectively equal to $c_{v_1} = 8\%$, $c_{v_2} = 17\%$, $c_{v_3} = 41\%$ and $c_{v_4} = 62\%$. For each group, 10 trials of duration $T = 30s$ were run, with $c = 1$ and initial conditions equal to $\theta_k(0) = \frac{\pi}{2}\ \forall k \in [1, N]$.

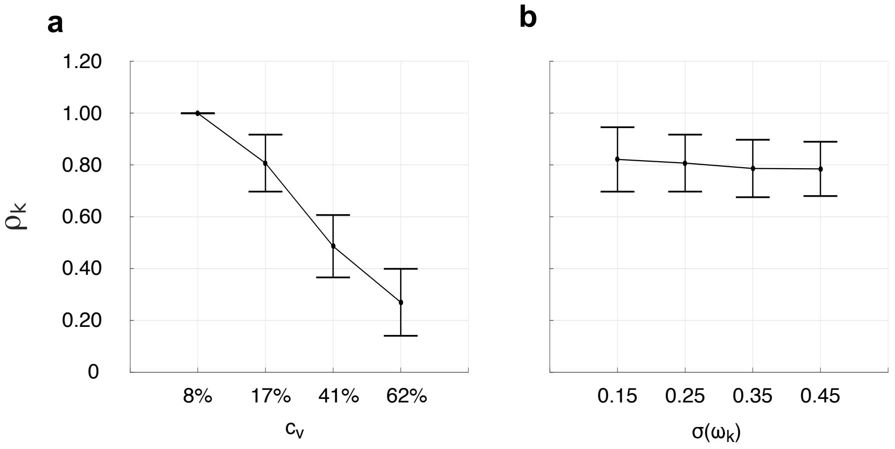

Supplementary Figure 10: **Individual synchronisation indices ρ_k as a function of overall dispersion c_v and intra-individual variability $\sigma(\omega_k)$ of the natural oscillation frequencies.** Individual synchronisation indices are shown for the four groups with equal $\sigma(\omega_k) = 0.25$ and different c_v (**a**), as well as for those with equal $c_v = 17\%$ and different $\sigma(\omega_k)$ (**b**). Mean values over the total number of nodes are represented by circles, and standard deviations by error bars.

Supplementary Fig. 10a shows the values of the individual synchronisation indices ρ_k as a function of the overall dispersion c_v. A One-way ANOVA using the Welch's test revealed a statistically significant effect of c_v ($F(3, 10) = 109.345$, $p < 0.001$, $\eta^2 = 0.896$). A post-hoc multiple comparison using the Games-Howell test is detailed in Supplementary Table 14, showing that the differences in ρ_k are statistically significant between all the groups ($p < 0.05$).

Secondly, we considered four other different groups of $N = 7$ heterogeneous Kuramoto oscillators. In order to isolate the effects of the intra-individual variability of the natural oscillation frequencies, the overall frequency dispersion was not varied over the groups ($c_v = 17\%$), whereas $\sigma(\omega_k)$ was varied across them ($\sigma(\omega_k) = 0.15$ for each kth node of the first group, $\sigma(\omega_k) = 0.25$ for the second group, $\sigma(\omega_k) = 0.35$ for the third group, $\sigma(\omega_k) = 0.45$ for the fourth group). The other parameters were set as in previous case.

Supplementary Table 14: **Post-hoc multiple comparisons – individual synchronisation indices ρ_k – effects of c_v**

c_v	**8%**	**17%**	**41%**	**62%**
**8%**	–	0.014	$\simeq 0$	$\simeq 0$
**17%**	0.014	–	0.001	$\simeq 0$
**41%**	$\simeq 0$	0.001	–	0.031
**62%**	$\simeq 0$	$\simeq 0$	0.031	–

Supplementary Fig. 10b shows the values of the individual synchronisation indices ρ_k as a function of the intra-individual variability $\sigma(\omega_k)$ of the natural oscillation frequencies. A One-way ANOVA revealed no statistically significant effect of $\sigma(\omega_k)$ ($F(3, 24) = 0.170$, $p = 0.916$, $\eta^2 = 0.019$).

Overall, these results suggest that, unlike the intra-individual variability of oscillation frequencies $\sigma(\omega_k)$, the overall dispersion c_v has a significant effect on the coordination levels of the group members.

8.2 Location of the link getting removed from a Ring graph

We then considered a network of $N = 7$ heterogeneous Kuramoto oscillators connected over a Path graph topology (Fig. 2c in the main text). Seven scenarios were considered, where each scenario differs from the others in the Ring graph connection (Fig. 2b in the main text) getting removed to form the Path graph itself. Specifically, in the first scenario the connection between nodes 1 and 2 was removed, in the second scenario that between nodes 2 and 3, up to the seventh scenario where the connection between nodes 7 and 1 was removed. In order to isolate the effects of such choice on the coordination levels ρ_k, the group dispersion was not varied over the different scenarios ($c_v = 17\%$), and neither were the individual variabilities across all the nodes ($\sigma(\omega_k) = 0.25\ \forall k \in [1, N]$). The other parameters were set as in the previous cases.

A One-way ANOVA revealed that the location of the link getting removed from a Ring graph to form a Path graph, and hence the difference between the frequencies of the nodes getting disconnected, does not have a significant effect on the coordination levels of its members ($F(6, 42) = 0.535$, $p = 0.778$, $\eta^2 = 0.071$). Mean values and standard deviations of the individual synchronisation index ρ_k obtained in the seven different scenarios here considered are shown in Supplementary Fig. 11.

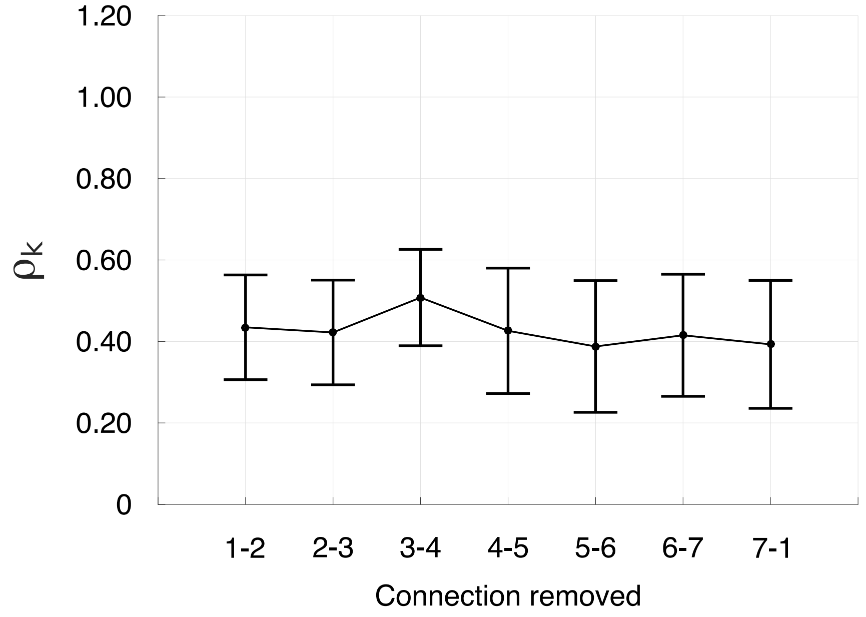

Supplementary Figure 11: **Individual synchronisation indices ρ_k as a function of the link getting removed in a Ring graph topology to form a Path Graph.** Mean values over the total number of nodes are represented by circles, and standard deviations by error bars.

8.3 Selection of the central node in a Star graph

We finally considered a network of $N = 7$ heterogeneous Kuramoto oscillators connected over a Star graph topology (Fig. 2d in the main text). Seven scenarios were considered, where each scenario differs from the others in the selection of the central node. Specifically, in the first scenario the central node was set to be node 1, in the second scenario node 2, up to the seventh scenario where the central node was set to be node 7. In order to isolate the effects of such choice on the coordination levels ρ_k, the group dispersion was not varied over the different scenarios ($c_v = 17\%$), and neither were the individual variabilities across all the nodes ($\sigma(\omega_k) = 0.25 \; \forall k \in [1, N]$). The other parameters were set as in the previous cases.

A One-way ANOVA using the Welch's test revealed that the differences in the coordination levels obtained for different choices of the central node in a Star graph topology are not statistically significant ($F(6, 18.367) = 0.463$, $p = 0.827$, $\eta^2 = 0.070$). Mean values and standard deviations of the individual synchronisation index ρ_k obtained in the seven different scenarios here considered are shown in Supplementary Fig. 12.

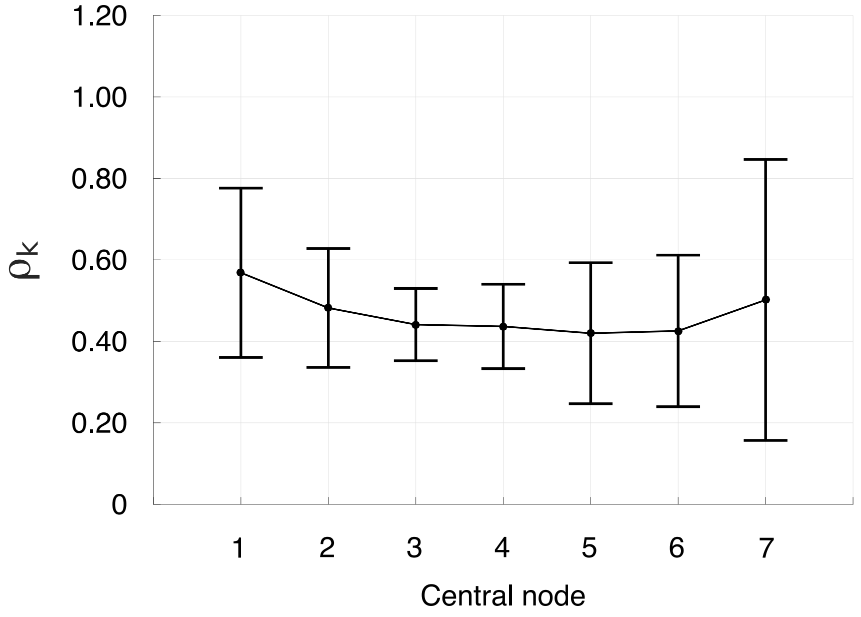

Supplementary Figure 12: **Individual synchronisation indices ρ_k as a function of the central node selection in a Star graph topology.** Mean values over the total number of nodes are represented by circles, and standard deviations by error bars.
